# Microbiological Factors and Patient-Reported Outcomes of Exacerbation Burden in *Pseudomonas aeruginosa*-Colonised Bronchiectasis: A One-Year Prospective Study

**DOI:** 10.3390/medsci14060596

**Published:** 2026-09-22

**Authors:** Leticia Bueno-Freire, Patricia Oscanoa-Huamán, Rubén López-Aladid, Victoria Alcaraz-Serrano, Nil Vázquez, Roberto Cabrera, Albert Gabarrús, Adrián Ceccato, Miquel Ferrer, Néstor Soler, Antoni Torres, Laia Fernández-Barat

**Affiliations:** 1 Institut d’Investigacions Biomèdiques August Pi i Sunyer (IDIBAPS)—Fundació Clínic per la Recerca Biomèdica (FCRB), CibeRes (Centro de Investigación Biomédica en Red de Enfermedades Respiratorias, 06/06/0028), Hospital Clínic de Barcelona, 08036 Barcelona, Spain; l.bueno@ieinstituto.es (L.B.-F.); patricia.oscanoa.h@gmail.com (P.O.-H.); ruben.aladid@gmail.com (R.L.-A.); nilsaxo@gmail.com (N.V.); rcabrera@recerca.clinic.cat (R.C.); gabarrus@recerca.clinic.cat (A.G.); adrianceccato@gmail.com (A.C.); miferrer@clinic.cat (M.F.); nsoler@clinic.cat (N.S.); 2 Department of Medicine, School of Medicine and Health Sciences, University of Barcelona, 08036 Barcelona, Spain; 3 Spanish Institute for Nursing Research, General Nursing Council of Spain, 28023 Madrid, Spain; 4 Consorci Sanitari Alt Penedès-Garraf (CSAPG), 08810 Barcelona, Spain; 5 Barcelona Institute for Global Health (ISGlobal), 08036 Barcelona, Spain; victoriaalcarazserrano@gmail.com; 6 Blanquerna School of Health Science, Ramon Llull University, 08022 Barcelona, Spain; 7 Critical Care Center, Hospital Universitari Parc Taulí, Institut d’Investigació i Innovació Parc Taulí (I3PT-CERCA), 08208 Sabadell, Spain; 8 Institut Clínic Respiratori, Hospital Clínic de Barcelona, 08036 Barcelona, Spain

**Keywords:** bronchiectasis, *Pseudomonas aeruginosa*, exacerbations, health-related quality of life, patient-reported outcomes, hospitalisation

## Abstract

**Background:** Exacerbations are key determinants of disease progression and impaired health-related quality of life (HRQoL) in non-cystic fibrosis bronchiectasis (BE). Despite the recognised association between *Pseudomonas aeruginosa* (PA) and worse outcomes, factors driving exacerbation burden remain poorly characterised. In patients with PA-colonised BE, we aimed to assess whether microbiological factors, including colonisation status and PA phenotype, together with HRQoL domains and respiratory symptoms, were associated with time to first exacerbation, exacerbation severity, and post-exacerbation changes in HRQoL. **Materials and Methods:** This prospective longitudinal study followed PA-colonised BE adults for 12 months with quarterly stable-state assessments focussing on time and severity of first exacerbation. HRQoL was measured by the Quality of Life Questionnaire-Bronchiectasis (QOL-B). Multivariable Cox, Firth penalised logistic, and ANCOVA models were fitted in R. **Results:** A total of 68 patients were included. During follow-up, 52 patients (76.5%) experienced ≥1 exacerbation, including 14 (26.9%) whose first event required hospitalisation. Neither colonisation status nor PA phenotype were associated with exacerbation risk or hospitalisation. Better daytime cough (HR 0.63, 95%CI 0.41–0.97; *p* = 0.036) and dyspnoea while talking (HR 0.47, 95%CI 0.26–0.87; *p* = 0.016) were independently associated with lower exacerbation risk. Lower exertional dyspnoea burden (OR 0.29, 95%CI 0.10–0.67; *p* = 0.003) and lower sputum quantity (OR 0.46, 95%CI 0.19–0.93; *p* = 0.030) were associated with lower odds of hospitalisation, together with poorer Vitality, Social Functioning, and Health Perceptions. After exacerbation, Social Functioning declined significantly (Δ −7.50 points, 95%CI −14.87 to −0.14; *p* = 0.046) and mucoid PA was independently associated with worse Emotional Functioning (β −15.64, 95%CI −27.89 to −3.39; *p* = 0.013). **Conclusions:** In PA-colonised BE, selected respiratory symptoms and HRQoL domains were associated with exacerbation burden, whereas microbiological characteristics showed no clear associations with exacerbation risk or hospitalisation, supporting the incorporation of patient-reported outcomes into longitudinal assessment.

## 1. Introduction

Exacerbations in non-cystic fibrosis bronchiectasis (BE) represent pivotal events shaping disease progression, health-related quality of life (HRQoL), and healthcare-resource utilisation [1,2]. Prevention and effective management are essential to mitigating their long-term impact on disease trajectory, patient-reported outcomes, and healthcare costs [3,4,5,6]. Several factors contribute to exacerbation risk, including prior exacerbations, chronic airway infection, impaired mucociliary clearance, comorbidities, and heightened airway inflammation [7,8,9]. Symptom burden, as assessed by the Quality of Life–Bronchiectasis Respiratory Symptom Scale (QoL-B-RSS), has been shown to predict exacerbation and hospitalisation risk, emphasising the importance of patient-centred approaches to care [10,11].

Patients colonised with *Pseudomonas aeruginosa* (PA) face a particularly challenging disease course, characterised by frequent exacerbations, higher hospitalisation rates, accelerated lung function decline, and poorer HRQoL [12,13]. Persistent PA infection promotes biofilm formation, chronic airway inflammation, and antimicrobial resistance, perpetuating a cycle of recurrent infections and airway damage [12]. However, PA-colonised bronchiectasis remains clinically heterogeneous. In our cohort, chronic PA status was associated with higher systemic anti-*Pseudomonas* antibody levels, and this was associated with a lower number of hospitalised exacerbations observed during one year of follow-up. These findings suggest that the clinical implications of chronic PA colonisation depend not only on microbiological characteristics, but also on the host-immune response and its potential modulation of exacerbation severity [13,14]. This heterogeneity may also extend to patient-reported outcomes. While prior studies have demonstrated associations between the QoL-B-RSS and exacerbation risk in broader BE populations, less is known about the contribution of other QOL-B domains and individual respiratory symptoms specifically in this population [10,11]. In addition, data evaluating post-exacerbation changes in HRQoL, particularly according to exacerbation severity, remain limited [15].

Given the substantial impact of exacerbations, better understanding of microbiological and HRQoL dimensions may improve characterisation of exacerbation burden and support more patient-centred assessment. The primary aim of this study was to identify microbiological factors, HRQoL domains, and individual QoL-B-RSS associated with time to first exacerbation in patients with PA-colonised BE. Secondary aims were to explore the association of these factors with exacerbation severity and to evaluate changes in HRQoL following exacerbations.

## 2. Materials and Methods

### 2.1. Study Design and Participants

This prospective longitudinal observational study included adults with high-resolution computed tomography-confirmed BE and at least one PA-positive sputum culture obtained during stable disease within the year preceding enrolment. Patients were required to be clinically stable for ≥4 weeks before inclusion. Exclusion criteria comprised cystic fibrosis, sarcoidosis, diffuse interstitial lung disease, active tuberculosis, non-tuberculous mycobacterial infection, immunosuppression (except stable IgG replacement therapy), inability to comply with study procedures, or participation in clinical trials within the previous 6 months.

The study was approved by the Clinical Research Ethics Committee (HCB/2018/0236), and written informed consent was obtained from all participants.

### 2.2. Clinical Assessments and Follow-Up

Baseline assessments included age, sex, Bronchiectasis Severity Index (BSI), and Charlson Comorbidity Index (CCI). Baseline maintenance treatments were recorded from patients’ medical records and reflected routine clinical practice. These included long-term oral antibiotics, nebulised antibiotics, inhaled corticosteroids, long-acting bronchodilators (comprising long-acting β_2_-agonists [LABAs] and/or long-acting muscarinic antagonists [LAMAs]), and hypertonic saline. Spontaneous sputum samples were collected to determine PA colonisation status (intermittent vs. chronic), and phenotype (mucoid vs. non-mucoid). PA isolates were identified by MALDI-TOF, and the non-mucoid/mucoid phenotype was determined according to colony morphology, as previously described [16,17]. Chronic colonisation was defined as ≥2 consecutive PA-positive cultures separated by ≥3 months within 1 year, while intermittent colonisation involved alternating positive and negative cultures following initial infection [3,5].

After enrolment, patients were followed prospectively for one year with quarterly assessments during stable state, including HRQoL and exacerbation frequency and severity. Prior to each visit, clinical stability was confirmed through medical record review and telephone contact. After an exacerbation, this required completion of treatment followed by ≥4 weeks without further clinical deterioration. HRQoL was measured using the Quality of Life Questionnaire-Bronchiectasis (QOL-B) v3.1, a validated 37-item tool comprising eight domains standardised on a 0–100-point scale, with higher scores indicating better HRQoL [18,19]. Exacerbations were defined according to international guidelines as worsening of ≥3 symptoms for ≥48 h necessitating treatment changes [1] and classified as outpatient-managed or hospitalised.

### 2.3. Statistical Analysis

Time to first exacerbation was analysed using Kaplan–Meier curves and multivariable Cox proportional hazards models. Analyses evaluated microbiological factors (colonisation status and PA phenotype), eight QOL-B domains, and nine QoL-B-RSS items. Collinearity was assessed by correlation matrices and variance inflation factors before multivariable modelling. Microbiological variables were primarily modelled separately due to sparse subgroup distribution, whilst QOL-B domains and QoL-B-RSS items were additionally examined in mutually adjusted models. Two complementary adjustment strategies were applied. A severity-adjusted model included Bronchiectasis Severity Index (BSI) and sex, whereas a clinically adjusted model included age, FEV_1_ (% predicted), exacerbations in the previous year, and sex. Covariates were selected a priori based on clinical relevance and previous evidence regarding BE severity and exacerbation risk. QOL-B hazard ratios (HRs) were reported per 10-point increase in domain scores to facilitate clinical interpretation. The proportional hazards assumption was assessed using Schoenfeld residuals. The severity of the first exacerbation (ambulatory vs. hospitalised) was examined using multivariable Firth penalised logistic regression given the limited number of hospitalised events. Both individual and mutually adjusted models were examined; individual models provided the reported hospitalisation estimates, whereas mutually adjusted models were used as complementary analyses to assess the consistency of findings. Analyses applied the same modelling strategy and adjustment approach described above. Across the Cox and Firth analyses, individual models included three or six predictor degrees of freedom under the severity-adjusted and clinically adjusted strategies, respectively; the corresponding mutually adjusted QOL-B domain models included 10 and 13, and the QoL-B-RSS models included 11 and 14 predictor degrees of freedom, respectively. Post-exacerbation changes in QOL-B scores were evaluated using paired t-tests and mean changes (Δ = POST − PRE). Differences in post-exacerbation QOL-B scores according to microbiological characteristics were further examined using analysis of covariance (ANCOVA) adjusted for pre-exacerbation scores and exacerbation severity. Separate ANCOVA models evaluating exacerbation severity were adjusted for pre-exacerbation scores, BSI, and sex. All analyses were performed in R. Statistical significance was set at *p* < 0.05. Given the exploratory nature of the analyses and the limited sample size, no formal adjustment for multiple comparisons was applied and findings should be interpreted as hypothesis-generating.

## 3. Results

### 3.1. Baseline Characteristics and Follow-Up Outcomes

A total of 68 patients (45 females, mean age 71 ± 12 years) were included. Table 1 shows baseline characteristics stratified according to exacerbation status during follow-up. Patients who experienced exacerbations had significantly higher BSI scores and lower baseline Emotional and Social Functioning scores compared with non-exacerbators.

During the 12-month follow-up, 52 patients (76.5%) experienced at least one BE exacerbation, including 47 (69.1%) with at least one ambulatory event and 22 (32.4%) requiring hospitalisation. Notably, 17 patients (25%) experienced both ambulatory and hospitalised exacerbations during the year. The median time to first exacerbation was 4.3 months (IQR 2.2–11.1). Among patients with exacerbations, the first event was managed in the outpatient setting in 38 cases (73.1%), whereas 14 (26.9%) required hospital admission. Overall, four patients (5.9%) died during follow-up, with causes including BE exacerbation (*n* = 2), cardiac disease (*n* = 1), and cancer (*n* = 1).

### 3.2. Time to First Exacerbation

#### 3.2.1. Microbiological Factors

Neither chronic colonisation (adjusted HR 1.21, 95%CI 0.61–2.41; *p* = 0.581) nor mucoid phenotype (adjusted HR 0.96, 95%CI 0.55–1.67; *p* = 0.889) showed an association with time to first exacerbation. Complementary clinically adjusted models yielded similar results.

#### 3.2.2. Health-Related Quality of Life

In the mutually adjusted model including all QOL-B domains, no domain showed a statistically significant independent association with time to first exacerbation, although lower Social Functioning (HR 0.90 per 10-point increase, 95%CI 0.80–1.01; *p* = 0.064) and higher Treatment Burden (HR 1.14, 95%CI 0.98–1.34; *p* = 0.089) showed suggestive but non-significant associations with exacerbation risk. Similar findings were observed in individually adjusted models.

#### 3.2.3. Respiratory Symptoms

In the mutually adjusted QoL-B-RSS model, better daytime cough (HR 0.63, 95%CI 0.41–0.97; *p* = 0.036) and dyspnoea while talking scores (HR 0.47, 95%CI 0.26–0.87; *p* = 0.016) were associated with a lower hazard of first exacerbation (Figure 1). The association for daytime cough was consistent across individually adjusted models.

### 3.3. Severity of the First Exacerbation

Baseline characteristics according to the severity of the first exacerbation are shown in Supplementary Table S1. Time to first exacerbation did not differ according to its severity (adjusted HR 0.87, 95%CI 0.46–1.66; *p* = 0.669).

#### 3.3.1. Microbiological Factors

Among patients who experienced at least one exacerbation, neither chronic colonisation (adjusted OR 0.66, 95%CI 0.16–2.91; *p* = 0.574) nor mucoid phenotype (adjusted OR 1.22, 95%CI 0.34–4.45; *p* = 0.762) were associated with severity of the first episode. Similar findings were observed in the mutually adjusted microbiological model.

#### 3.3.2. Health-Related Quality of Life

None of the QOL-B domains were significantly associated with the severity of the first exacerbation, although better Vitality showed a borderline association with lower odds of hospitalisation (OR 0.80 per 10-point increase, 95%CI 0.61–1.01; *p* = 0.056). Similar findings were observed in the mutually adjusted QOL-B model. In clinically adjusted models better Vitality (OR 0.78, 95%CI 0.58–0.99; *p* = 0.040), Social Functioning (OR 0.82, 95%CI 0.64–1.00; *p* = 0.044) and Health Perceptions (OR 0.62, 95%CI 0.39–0.94; *p* = 0.021) were associated with lower odds of hospitalisation (Figure 2A; Supplementary Table S2).

#### 3.3.3. Respiratory Symptoms

Among individual QoL-B-RSS items, higher exertional dyspnoea scores, indicating lower symptom burden, were associated with lower odds of hospitalisation during the first exacerbation (OR 0.33 per one-point increase, 95%CI 0.14–0.66; *p* = 0.001). Sputum quantity showed a similar but borderline association (OR 0.52, 95%CI 0.24–1.03; *p* = 0.063). In clinically adjusted models, exertional dyspnoea remained associated with lower odds of hospitalisation (OR 0.29, 95%CI 0.10–0.67; *p* = 0.003), and sputum quantity became statistically significant (OR 0.46, 95%CI 0.19–0.93; *p* = 0.030) (Figure 2B; Supplementary Table S3).

### 3.4. Changes in HRQoL After the First Exacerbation

The mean ± SD time from the pre-exacerbation HRQoL assessment to the first exacerbation was 49.18 ± 17.64 days, and 52.91 ± 19.87 days from the first exacerbation to the subsequent stable-state assessment. Changes in HRQoL after the first exacerbation were generally small, with limited evidence of persistent deterioration at the subsequent stable state assessment except for a significant decline in Social Functioning (Δ −7.50 points; 95% CI −14.87 to −0.14; *p* = 0.046) (Supplementary Table S4). Regarding microbiological characteristics, Social Functioning exhibited greater decline in chronic colonised patients following exacerbation (Δ −8.87 points; 95% CI −16.67 to −1.07, *p* = 0.027). Additionally, among intermittently colonised patients, improvements in Vitality and Emotional Functioning and worsening in Treatment Burden exceeded the proposed minimal clinically important difference (MCID) thresholds, although none of these changes reached statistical significance (Supplementary Table S5). Differences by PA phenotype were not significant, but mucoid PA showed declines exceeding MCID in Emotional Functioning (Supplementary Table S6). Larger declines were observed following hospitalised exacerbations, particularly in Social Functioning and Treatment Burden, where changes exceeded MCID despite not reaching statistical significance (Supplementary Table S7). These descriptive post-exacerbation changes according to colonisation status, PA phenotype, and exacerbation severity are shown in Figure 3.

In ANCOVA models, mucoid PA phenotype was associated with worse Emotional Functioning following exacerbation (β −15.64, 95% CI −27.89 to −3.39, *p* = 0.013). Colonisation status and severity of the exacerbation were not significantly associated with worse post-exacerbation scores in any QOL-B domain, although larger declines were observed.

## 4. Discussion

This prospective study evaluated microbiological characteristics, HRQoL, and respiratory symptoms associated with exacerbation burden in PA-colonised BE patients over one year. Microbiological characteristics and QOL-B domains were not related to time to first exacerbation, whereas daytime cough and dyspnoea while talking were linked to earlier exacerbation. Among patients who experienced at least one exacerbation, poorer Vitality, Social Functioning and Health Perceptions, together with greater dyspnoea and sputum quantity, were associated with higher odds of hospitalisation. Changes in HRQoL following exacerbation were generally small, although Social Functioning declined significantly, and chronic colonisation, mucoid PA, and hospitalised exacerbations showed greater deterioration across Emotional, Social Functioning and Treatment Burden domains.

In our cohort, time to first exacerbation appeared to be driven more by baseline clinical severity and symptomatic instability rather than by microbiological characteristics or QOL-B domains. Although PA infection is consistently related to greater exacerbation burden in broader BE populations, this evidence has largely arisen from comparisons between PA-colonised and non-colonised patients [12,13,20]. The relevance of sputum microbiome composition in BE has been highlighted by several authors. Dicker et al. showed that clinically stable patients clustered according to dominant microbial profiles and that these profiles were associated with long-term outcomes. In particular, dominance by classical pathogenic taxa, such as Pseudomonas or Stenotrophomonas, was associated with poorer survival than other microbiome profiles [21]. The authors also reported an apparently counterintuitive association between PA- and *Haemophilus*-dominated profiles and clinical stability, despite the conventional use of these taxa to support the identification of bacterial exacerbations. Other microbiome studies have similarly reported a greater relative abundance of PA among patients experiencing fewer hospitalised exacerbations [22]. Finally, an integrative analysis of our cohort in Barcelona showed that chronic PA infection was associated with higher systemic anti-PA antibody levels, which were proposed as a potential explanation for the lower number of hospitalised exacerbations observed in these patients during one year of follow-up [14,23]. Collectively, these findings suggest that conventional stratification according to colonisation status or non-mucoid/mucoid phenotype may not fully capture the complex microbial and host factors associated with exacerbation burden. In the present study, neither colonisation status nor non-mucoid/mucoid phenotype was independently associated with time to first exacerbation or with the severity of the first exacerbation after adjustment for disease severity. Within an exclusively PA-colonised cohort, baseline clinical severity may therefore be more informative for short-term exacerbation risk than further stratification according to colonisation chronicity or non-mucoid/mucoid phenotype. However, clinically relevant effects cannot be excluded given the limited sample size and wide confidence intervals. Similarly, although several QOL-B domains showed directional trends towards earlier exacerbation, these associations were attenuated after adjustment for disease severity. While poorer QOL-B scores have previously been associated with greater disease burden and worse clinical status [18,19], our results suggest that global HRQoL impairment may largely mirror cumulative disease burden in PA-colonised populations rather than providing independent prognostic information regarding short-term exacerbation risk. Unlike larger general BE cohorts, in which the overall QoL-B-RSS and individual symptoms predicted exacerbation risk [10,11], our exclusively PA-colonised cohort exhibited a more selective prognostic signal primarily involving cough and dyspnoea, while other symptoms showed directional but non-significant associations. These findings suggest that daily respiratory symptoms may capture dynamic airway dysfunction and clinical instability more directly than broader HRQoL domains or microbiological characteristics alone.

Regarding severity of the first exacerbation, neither colonisation status nor non-mucoid/mucoid phenotype were linked to hospitalisation risk. Although chronic and mucoid PA have been linked to poorer clinical outcomes in PA-colonised BE [14,23,24], our findings suggest that these microbiological characteristics may be less informative for discriminating exacerbation severity. Indeed, we previously reported significantly higher levels of circulating anti-*Pseudomonas* antibodies in chronically PA-colonised patients with bronchiectasis, which could potentially modulate the clinical expression and severity of subsequent exacerbations [14]. By contrast, patients with poorer Vitality, Social Functioning and Health Perceptions, together with worse exertional dyspnoea and sputum quantity, were associated with hospitalised exacerbations. These domains and symptoms may better reflect the functional and respiratory vulnerability underlying severe exacerbations than microbiological characteristics alone. Previous studies have linked impaired HRQoL and respiratory symptom burden to increased exacerbation risk and worse clinical outcomes in BE [10,11,13,20], and our findings suggest that these assessments may also help identify patients at greater risk of severe exacerbations within PA-colonised disease.

Post-exacerbation changes in HRQoL were generally modest across QOL-B domains, although Social Functioning declined significantly, suggesting that selected psychosocial dimensions may remain affected despite apparent return to clinical stability. This is consistent with previous evidence showing that the impact of BE exacerbations on HRQoL may persist beyond physiological recovery [15]. In patients with chronic colonisation, Social Functioning declined significantly. By contrast, in patients with intermittent colonisation, improvement in Vitality and Emotional Functioning and worsening in Treatment Burden exceeded the proposed MCID thresholds [18]; however, these differences were not confirmed in multivariable models. These findings suggest that exploratory domain-specific patterns of post-exacerbation HRQoL in our cohort were attenuated after accounting for baseline disease severity. Although chronic PA colonisation is recognised as a marker of a more severe clinical phenotype, its association with post-exacerbation HRQoL in our cohort was attenuated after accounting for baseline disease severity, aligning with prior evidence linking chronic PA colonisation to poorer clinical outcomes and HRQoL [20,25]. By contrast, post-exacerbation Emotional Functioning exceeded MCID in patients with mucoid PA and was independently associated with worse scores, suggesting that more complex microbiological phenotypes may contribute to greater emotional vulnerability and perceived disease impact following exacerbation. This is in line with evidence relating mucoid PA to impaired mucus clearance, altered sputum viscoelasticity, and more severe clinical outcomes [23]. Hospitalised exacerbations also showed MCID-exceeding declines in Social Functioning and Treatment Burden, suggesting that more severe events may have a greater impact on patients’ social participation and perceived treatment demands. This is consistent with previous evidence linking severe exacerbations and hospitalisation burden to worse HRQoL [2,7,20]. Although several subgroup differences did not reach statistical significance, the consistency of MCID-exceeding declines across psychosocial and treatment-related domains supports the potential clinical relevance of these findings. Together, these findings indicate that in PA-colonised BE the impact of exacerbations may be modest at subsequent stable-state assessment, but selected subgroups—particularly patients with mucoid PA, chronic colonisation, or hospitalised exacerbations—may experience clinically meaningful deterioration after exacerbation in psychosocial and treatment-related domains. Moreover, a longer follow-up may be needed to capture changes attributable to colonisation status and their potential impact on clinical outcomes.

These findings reinforce the importance of incorporating patient-reported outcomes into longitudinal monitoring in PA-colonised BE. The associations observed between QOL-B domains, Respiratory Symptoms and exacerbation burden suggest that these measures may help identify patients at increased risk of early or severe exacerbations beyond microbiological assessment alone. In this context, these findings may help inform future nursing-led monitoring and self-management strategies aimed at recognising symptom deterioration, supporting treatment adherence and facilitating earlier intervention during clinical worsening [26,27,28]. Routine incorporation of HRQoL and symptom assessment into longitudinal follow-up may therefore contribute to a more personalised approach to exacerbation prevention and management.

This study has several strengths. Its prospective longitudinal design with quarterly follow-up enabled detailed assessment of exacerbation risk, severity, and post-exacerbation HRQoL changes in a well-characterised cohort of PA-colonised BE patients. The exclusive inclusion of this population allowed focused evaluation of clinically relevant PA characteristics, including colonisation status and phenotype. The use of validated definitions and instruments, together with analyses of all QOL-B domains and individual QoL-B-RSS items, strengthened the reliability of the findings and provided a patient-centred evaluation of exacerbation burden. In addition, robust statistical approaches, including complementary adjustment strategies and penalised regression models, enhanced estimate reliability and reduced the risk of small-sample bias. However, several limitations should also be acknowledged. First, the single-centre design and moderate sample size may limit generalisability and statistical power, particularly for subgroup analyses. Nonetheless, this setting ensures consistency in clinical protocols and population characteristics, potentially reducing variability from site-specific factors. Second, the observational design precludes causal inference, and residual confounding cannot be excluded despite multivariable adjustment. Third, maintenance treatments were heterogeneous and were not included in the prespecified adjustment strategy; therefore, residual confounding by treatment indication cannot be excluded, as these treatments may influence exacerbation burden, respiratory symptoms, airway microbiology, and HRQoL. In addition, the relatively large number of exploratory comparisons performed in relation to the sample size increases the possibility of chance findings, particularly for associations with *p*-values close to the conventional significance threshold, including the post-exacerbation decline in Social Functioning (*p* = 0.046). As no formal adjustment for multiple comparisons was applied, these findings should be considered hypothesis-generating and require confirmation in larger independent cohorts or registries. With 52 first-exacerbation events, the mutually adjusted Cox models may be susceptible to model instability and overfitting. This concern is greater for the logistic regression analyses of first-exacerbation severity, which included only 14 hospitalisations. Accordingly, estimates from the mutually adjusted Cox models and the hospitalisation analyses should be interpreted as exploratory. Furthermore, PA phenotypic characterisation focused on the non-mucoid/mucoid phenotype, given the clinical implications of this distinction reported in previous studies, but did not capture the broader phenotypic diversity or within-patient heterogeneity of PA. Finally, the exclusive focus on PA-colonised patients, while intentional, limits applicability to other pathogens or non-colonised populations. Future studies should prioritise larger multicentre cohorts with longer follow-up, incorporating more comprehensive PA phenotypic characterisation and lung microbiome profiling, to better characterise the long-term exacerbation burden and patient-reported outcomes in PA-colonised BE.

## 5. Conclusions

In PA-colonised BE, additional microbiological stratification showed limited value for distinguishing time to first exacerbation and its severity beyond the underlying clinical burden of disease. By contrast, respiratory symptoms related to cough, dyspnoea, and sputum production, together with selected HRQoL domains—particularly psychosocial dimensions—were more closely associated with exacerbation occurrence and hospitalisation risk. Although overall post-exacerbation changes in HRQoL were modest, Social Functioning declined at the subsequent stable-state assessment, and mucoid PA phenotype was independently associated with poorer post-exacerbation Emotional Functioning. Together, these findings reinforce the value of incorporating patient-reported outcomes and symptom tracking into the longitudinal monitoring of PA-colonised BE.

## Figures and Tables

**Figure 1:**
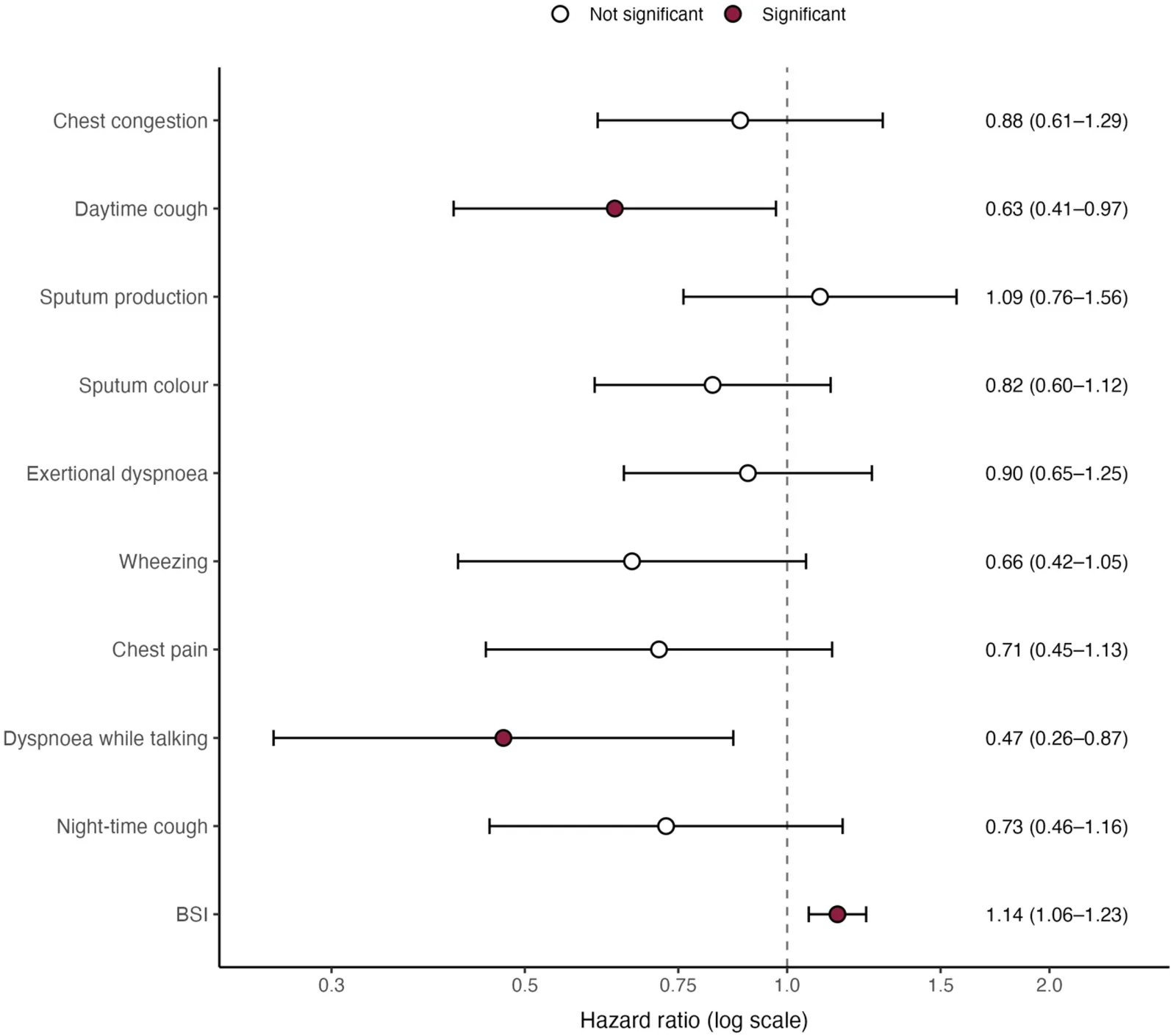
Associations of baseline respiratory symptoms and time to first exacerbation (*n* = 68). Forest plot shows hazard ratios (HRs) and 95% confidence intervals (95%CIs) from a multivariable Cox proportional hazards model including all individual items from the Respiratory Symptoms domain of the Quality of Life Questionnaire–Bronchiectasis (QOL-B), adjusted for Bronchiectasis Severity Index (BSI) and sex. These items range from one to four points, with higher scores indicating lower symptom burden; therefore, HRs below one indicate lower exacerbation risk associated with lower symptom burden. Filled circles represent statistically significant associations (*p* < 0.05).

**Figure 2:**
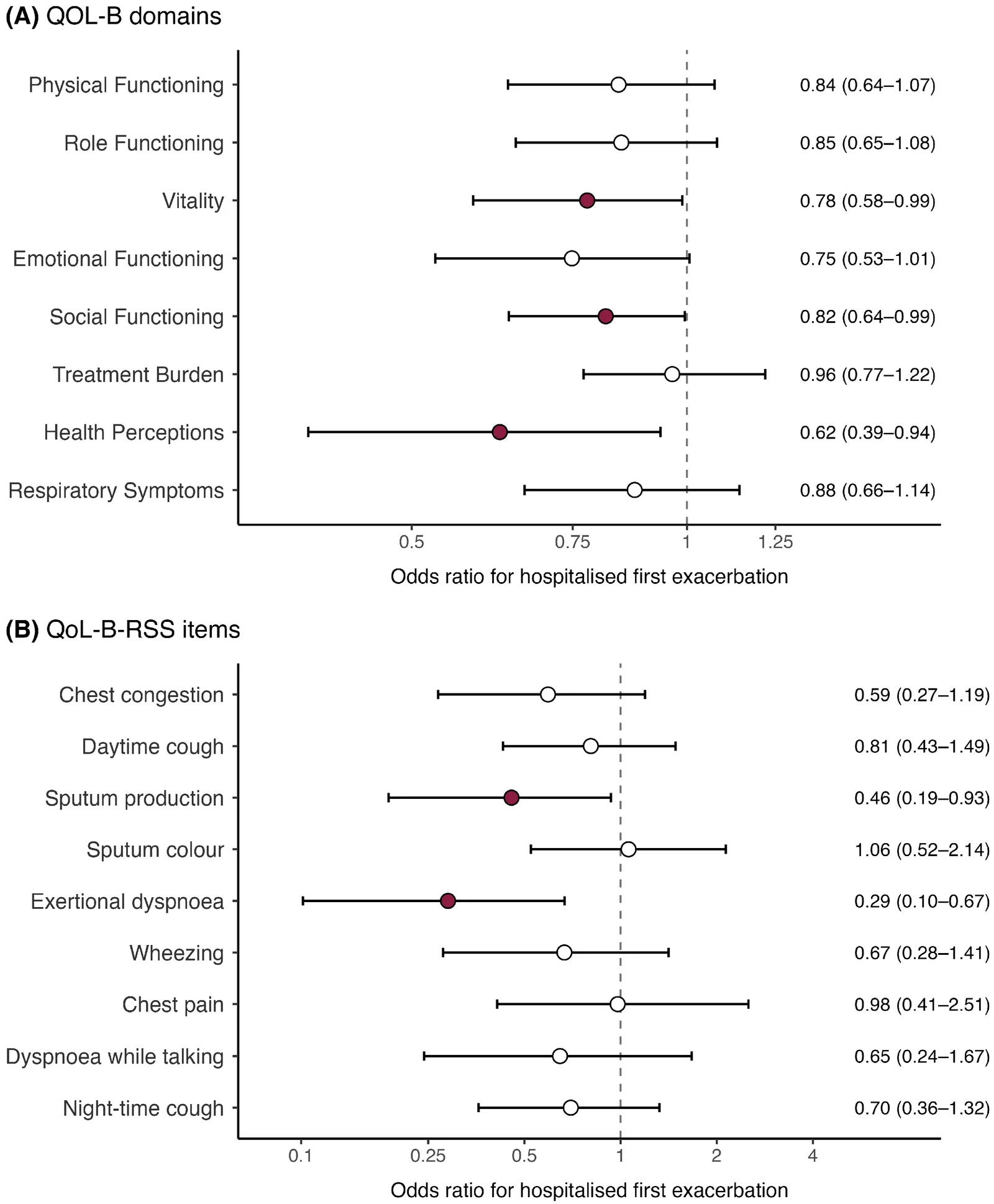
Associations of baseline health-related quality of life and respiratory symptoms with hospitalised first exacerbation (*n* = 52). Forest plots display odds ratios (ORs) and 95% confidence intervals (95%CIs) from multivariable Firth penalised logistic regression models evaluating associations between baseline Quality-of-Life Questionnaire–Bronchiectasis (QOL-B) domains (**Panel A**) and individual Respiratory Symptoms Scale (QoL-B-RSS) items (**Panel B**) with risk of hospitalised first exacerbation. Models were adjusted for age, FEV_1_ (% predicted), exacerbation frequency in the previous year, and sex. QOL-B domains are expressed per 10-point increase and QoL-B-RSS items per one-point increase, with higher scores indicating better HRQoL or lower symptom burden. Therefore, ORs below one indicate lower odds of hospitalisation associated with better baseline status. Filled circles represent statistically significant associations (*p* < 0.05).

**Figure 3:**
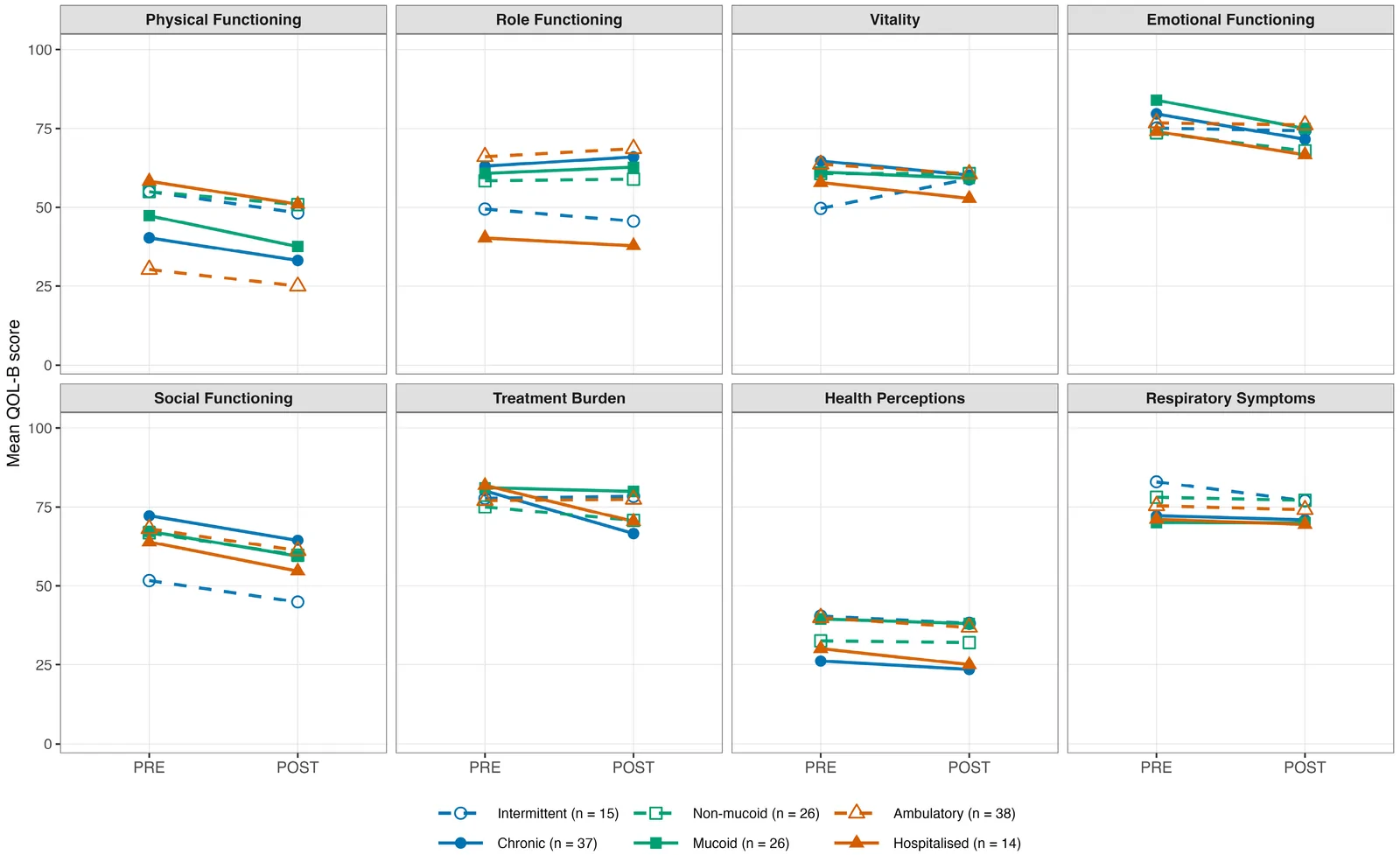
Post-exacerbation changes in health-related quality of life (HRQoL) according to microbiological characteristics and first exacerbation severity (*n* = 52). Mean pre-exacerbation (PRE) and post-exacerbation (POST) Quality of Life Questionnaire–Bronchiectasis (QOL-B) domain scores are shown according to colonisation status [intermittent (*n* = 11) vs. chronic (*n* = 41)], *Pseudomonas aeruginosa* (PA) phenotype [non-mucoid (*n* = 26) vs. mucoid (*n* = 26)], and exacerbation severity [ambulatory (*n* = 38) vs. hospitalised (*n* = 14)]. The mean ± SD interval from pre-exacerbation assessment to exacerbation was 49.18 ± 17.64 days and 52.91 ± 19.87 days from exacerbation to post-exacerbation assessment. Higher QOL-B scores indicate better HRQoL. Dashed lines represent intermittent colonisation, non-mucoid phenotype, and ambulatory exacerbations, whereas solid lines represent chronic colonisation, mucoid phenotype, and hospitalised exacerbations. Panels display descriptive mean changes across QOL-B domains following the first exacerbation during follow-up. Thus, differences between groups and changes over time should be interpreted cautiously, particularly for smaller subgroups.

**Table 1:** Baseline characteristics by exacerbation during follow-up (*n* = 68).

	No Exacerbation (*n* = 16)	Exacerbation (*n* = 52)	*p*-Value
Demographic and clinical characteristics			
Age, years	70.94 (8.72)	70.79 (13.05)	0.958
Sex			0.227
Male	3 (18.8%)	20 (38.5%)	
Female	13 (81.3%)	32 (61.5%)	
Bronchiectasis Severity Index (0–25)	9.00 (3.56)	12.44 (3.98)	0.003 *
Charlson Comorbidity Index (0–39)	2.50 (1.90)	2.77 (2.17)	0.636
Psychiatric and/or psychological conditions	6 (37.5%)	13 (25.0%)	0.353
FEV1 (% predicted)	65.67 (15.36)	60.61 (21.14)	0.303
Chronic treatment			
Long-term oral antibiotics	2 (12.5%)	20 (38.5%)	0.102
Nebulised antibiotics	1 (6.3%)	14 (26.9%)	0.162
Inhaled corticosteroids	6 (37.5%)	41 (78.8%)	0.005 *
Long-acting bronchodilators (LABA and/or LAMA)	12 (75.0%)	47 (90.4%)	0.244
Hypertonic saline	0 (0.0%)	5 (9.6%)	0.459
Microbiological characteristics			
Colonisation status			1.000
Intermittent	4 (25.0%)	11 (21.2%)	
Chronic	12 (75.0%)	41 (78.8%)	
PA phenotype			1.000
Non-mucoid	8 (50.0%)	26 (50.0%)	
Mucoid	8 (50.0%)	26 (50.0%)	
Health-related quality of life (QOL-B)			
Physical Functioning (0–100)	50.00 (29.81)	38.46 (29.06)	0.186
Role Functioning (0–100)	68.75 (22.67)	58.01 (29.05)	0.133
Vitality (0–100)	58.33 (25.82)	55.45 (29.65)	0.709
Emotional Functioning (0–100)	85.42 (15.96)	72.12 (25.93)	0.017 *
Social Functioning (0–100)	81.25 (22.67)	62.82 (35.79)	0.019 *
Treatment Burden (0–100)	88.46 (20.84)	77.08 (28.48)	0.121
Health Perceptions (0–100)	38.54 (20.83)	35.58 (19.81)	0.619
Respiratory Symptoms (0–100)	79.17 (20.64)	72.12 (25.08)	0.266
Chest congestion (1–4)	3.50 (0.89)	3.15 (0.96)	0.195
Daytime cough (1–4)	3.06 (0.68)	2.48 (1.08)	0.014 *
Sputum production (1–4)	2.81 (0.83)	2.38 (0.99)	0.097
Sputum colour (1–4)	2.69 (0.87)	2.62 (0.97)	0.781
Exertional dyspnoea (1–4)	3.13 (0.89)	2.73 (1.03)	0.145
Wheezing (1–4)	1.63 (0.89)	1.81 (0.86)	0.475
Chest pain (1–4)	3.81 (0.40)	3.52 (0.73)	0.045 *
Dyspnoea while talking (1–4)	1.56 (0.63)	1.54 (0.67)	0.896
Night-time cough (1–4)	3.56 (0.63)	3.15 (1.06)	0.064

Table 1 shows baseline demographic, clinical, microbiological, and health-related quality-of-life (HRQoL) characteristics stratified according to the presence of bronchiectasis exacerbation during the 12-month follow-up. Values are presented as mean (SD) or n (%). HRQoL was assessed using the Quality of Life Questionnaire-Bronchiectasis (QOL-B) v3.1. QOL-B domains are standardised on a 0–100 scale, with higher scores indicating better HRQoL. A total score integrating all domains is not calculated. Individual Respiratory Symptom items were scored from one to four, with higher scores indicating lower symptom burden. *p*-values were calculated using Welch’s two-sample t-test, Pearson’s chi-squared test, or Fisher’s exact test, as appropriate. * *p* < 0.05. *Abbreviations:* FEV_1_, *forced expiratory volume in one second*; PA, *Pseudomonas aeruginosa*; QOL-B, Quality of Life Questionnaire–Bronchiectasis.

## Data Availability

The original contributions presented in this study are included in the article/Supplementary Material. Further inquiries can be directed to the corresponding authors.

## Supplementary Materials

The following supporting information can be downloaded at: https://www.mdpi.com/article/10.3390/medsci14060596/s1: Table S1: Baseline characteristics by first exacerbation severity (*n* = 52); Table S2: Association of baseline QOL-B domains and severity of the first exacerbation (*n* = 52); Table S3: Association of baseline QOL-B Respiratory Symptoms and severity of the first exacerbation (*n* = 52); Table S4: Post-exacerbation changes in QOL-B domains (*n* = 52); Table S5: Post-exacerbation changes in QOL-B domains by colonisation status (*n* = 52); Table S6: Changes in QOL-B domains by *Pseudomonas aeruginosa* phenotype following the first exacerbation (*n* = 52); Table S7: Post-exacerbation changes in QOL-B domains by first exacerbation severity (*n* = 52).
