# Supplementary material for: Microbiological Factors and Patient-Reported Outcomes of Exacerbation Burden in Pseudomonas aeruginosa-Colonised Bronchiectasis: A One-Year Prospective Study
Source: Med Sci (Basel). 2026 Sep 22;14(6):596. doi: 10.3390/medsci14060596 (PMC13706908; doi:10.3390/medsci14060596)
Supplement: Supplementary file 1 [file medsci-14-00596-s001.zip › medsci-4520092-supplementary.pdf]

Supplementary material

Table S1. Baseline characteristics by first exacerbation severity (n = 52).

	Ambulatory (n = 38)	Hospitalised (n = 14)	P-value
Demographic and clinical characteristics			
**Age, years**	69.79 (13.38)	73.50 (12.16)	0.351
**Sex**			0.941
Male	14 (36.8%)	6 (42.9%)	
Female	24 (63.2%)	8 (57.1%)	
**Bronchiectasis Severity Index (0-25)**	11.63 (3.84)	14.64 (3.63)	0.015*
**Charlson Comorbidity Index (0-39)**	2.66 (2.16)	3.07 (2.27)	0.561
**Psychiatric and/or psychological conditions**	9 (23.7%)	4 (28.6%)	0.729
**FEV1 (% predicted)**	64.65 (20.75)	49.65 (18.72)	0.020*
Chronic treatment			
**Long-term oral antibiotics**	16 (42.1%)	4 (28.6%)	0.570
**Nebulised antibiotics**	12 (31.6%)	2 (14.3%)	0.371
**Corticosteroids**	31 (81.6%)	10 (71.4%)	0.680
**Long-acting bronchodilators (LABA and/or LAMA)**	33 (86.8%)	14 (100.0%)	0.370
**Hypertonic saline**	3 (7.9%)	2 (14.3%)	0.870
Microbiological characteristics			
**Colonisation status**			0.460
Intermittent	7 (18.4%)	4 (28.6%)	
Chronic	31 (81.6%)	10 (71.4%)	
**PA phenotype**			1.000
Non-mucoid	19 (50.0%)	7 (50.0%)	
Mucoid	19 (50.0%)	7 (50.0%)	
Health-related quality of life (QOL-B)			
**Physical Functioning (0-100)**	44.74 (29.28)	21.43 (21.11)	0.003*
**Role Functioning (0-100)**	62.28 (30.19)	46.43 (22.81)	0.051
**Vitality (0-100)**	60.96 (29.07)	40.48 (26.73)	0.024*
**Emotional Functioning (0-100)**	75.00 (26.49)	64.29 (23.44)	0.170
**Social Functioning (0-100)**	67.54 (35.50)	50.00 (34.59)	0.120
**Treatment Burden (0-100)**	78.10 (25.49)	74.36 (36.40)	0.738
**Health Perceptions (0-100)**	38.16 (18.95)	28.57 (21.11)	0.150
**Respiratory Symptoms (0-100)**	73.25 (25.57)	69.05 (24.33)	0.591
Chest congestion (1-4)	3.24 (0.94)	2.93 (1.00)	0.327
Daytime cough (1-4)	2.53 (1.08)	2.36 (1.08)	0.622
Sputum production (1-4)	2.53 (0.95)	2.00 (1.04)	0.112
Sputum colour (1-4)	2.53 (0.92)	2.86 (1.10)	0.328
Exertional dyspnoea (1-4)	3.05 (0.93)	1.86 (0.77)	<0.001*
Wheezing (1-4)	1.87 (0.93)	1.64 (0.63)	0.328
Chest pain (1-4)	3.50 (0.76)	3.57 (0.65)	0.739
Dyspnoea while talking (1-4)	1.50 (0.65)	1.64 (0.74)	0.533
Night-time cough (1-4)	3.18 (1.09)	3.07 (1.00)	0.727

Table S1 describes baseline demographic, clinical, microbiological, and health-related quality of life (HRQoL) characteristics according to the severity of the first bronchiectasis exacerbation during follow-up. Values are presented as mean (SD) or n (%). HRQoL was assessed using the Quality of Life Questionnaire-Bronchiectasis (QOL-B) v3.1. QOL-B domains are standardised on a 0–100 scale, with higher scores indicating better HRQoL. A total score is not calculated. Individual Respiratory Symptom items were scored from 1 to 4, with higher scores indicating lower symptom burden. P-values

were calculated using Welch's two-sample t-test, Pearson's chi-squared test, or Fisher's exact test, as appropriate. * $P < 0.05$. *Abbreviations:* FEV_1, *forced expiratory volume in one second;* PA, *Pseudomonas aeruginosa*; QOL-B, Quality of Life Questionnaire-Bronchiectasis.

Table S2. Association of baseline QOL-B domains and severity of the first exacerbation (n = 52).

	Main model OR (95%CI), p	Sensitivity analysis OR (95%CI), p
**Physical Functioning**	0.81 (0.61–1.05), p=0.111	0.84 (0.64–1.07), p=0.167
**Role Functioning**	0.88 (0.70–1.11), p=0.284	0.85 (0.65–1.08), p=0.181
**Vitality**	0.80 (0.61–1.01), p=0.056	0.78 (0.58–0.99), p=0.040*
**Emotional Functioning**	0.89 (0.69–1.12), p=0.317	0.75 (0.53–1.01), p=0.056 ·
**Social Functioning**	0.87 (0.72–1.04), p=0.130	0.82 (0.64–1.00), p=0.044*
**Treatment Burden**	0.94 (0.76–1.18), p=0.600	0.96 (0.77–1.22), p=0.746
**Health Perceptions**	0.82 (0.59–1.12), p=0.218	0.62 (0.39–0.94), p=0.021*
**Respiratory Symptoms**	0.89 (0.68–1.16), p=0.385	0.88 (0.66–1.14), p=0.328

Table S2 displays association between baseline health-related quality of life (HRQoL) domains and severity of the first bronchiectasis exacerbation during follow-up (n=52). Odds ratios (ORs) and 95% confidence intervals (95%CIs) were estimated using penalised logistic regression models with Firth correction. Main models were adjusted for Bronchiectasis Severity Index (BSI) and sex. Sensitivity analyses included age, FEV_1 (% predicted), frequent exacerbator status, and sex instead of BSI. HRQoL was assessed using the Quality of Life Questionnaire-Bronchiectasis (QOL-B) v3.1. Domains are standardised on a 0–100 scale, with higher scores reflecting better HRQoL; therefore, ORs <1 indicate lower odds of hospitalisation associated with better HRQoL. ORs are expressed per 10-point increase in the corresponding QOL-B domain score. *P <0.05.

Table S3. Association of baseline QOL-B Respiratory Symptoms and severity of the first exacerbation (n = 52).

	Main model OR (95%CI), p	Sensitivity analysis OR (95%CI), p
**Chest congestion**	0.59 (0.28–1.14), p=0.118	0.59 (0.27–1.19), p=0.145
**Daytime cough**	0.82 (0.44–1.47), p=0.500	0.81 (0.43–1.49), p=0.489
**Sputum production**	0.52 (0.24–1.03), p=0.063	0.46 (0.19–0.93), p=0.030*
**Sputum colour**	0.78 (0.40–1.49), p=0.454	0.94 (0.47–1.92), p=0.869
**Exertional dyspnoea**	0.33 (0.14–0.66), p=0.001*	0.29 (0.10–0.67), p=0.003*
**Wheezing**	0.66 (0.28–1.36), p=0.266	0.67 (0.28–1.41), p=0.302
**Chest pain**	0.96 (0.40–2.52), p=0.924	0.98 (0.41–2.51), p=0.963
**Dyspnoea while talking**	0.67 (0.26–1.69), p=0.382	0.65 (0.24–1.67), p=0.362
**Night-time cough**	0.88 (0.49–1.63), p=0.669	0.70 (0.36–1.32), p=0.266

Table S3 shows association between baseline respiratory symptoms and severity of the first bronchiectasis exacerbation during follow-up (n=52). Odds ratios (ORs) and 95% confidence intervals (95%CIs) were estimated using penalised logistic regression models with Firth correction. Main models were adjusted for Bronchiectasis Severity Index (BSI) and sex. Sensitivity analyses included age, FEV_1 (% predicted), exacerbations last year, and sex instead of BSI. Respiratory Symptoms were assessed using the Respiratory Symptoms domain of the Quality of Life Questionnaire-Bronchiectasis (QOL-B) v3.1. Items are standardised on a 1–4 scale, with higher scores reflecting less symptoms; therefore, ORs <1 indicate lower odds of hospitalisation associated with less symptom burden. *P <0.05.

Table S4. Post-exacerbation changes in QOL-B domains (n = 52).

QOL-B Domain	PRE (mean ± SD)	POST (mean ± SD)	P-value	Delta (95% CI)
**Physical Functioning**	51.29 ± 30.30	44.49 ± 31.00	0.061	-6.81 (-13.93 to 0.32)
**Role Functioning**	59.63 ± 29.55	60.91 ± 31.52	0.689	1.28 (-5.12 to 7.68)
**Vitality**	60.93 ± 29.67	59.91 ± 26.22	0.812	-1.03 (-9.69 to 7.63)
**Emotional Functioning**	74.25 ± 27.37	75.61 ± 25.41	0.720	1.36 (-6.22 to 8.93)
**Social Functioning**	67.04 ± 33.20	59.54 ± 29.73	0.046*	-7.50 (-14.87 to -0.14)
**Treatment Burden**	78.27 ± 30.74	75.63 ± 25.79	0.607	-2.64 (-12.93 to 7.65)
**Health Perceptions**	36.17 ± 22.30	35.15 ± 20.76	0.784	-1.02 (-8.43 to 6.40)
**Respiratory Symptoms**	73.90 ± 24.80	73.38 ± 21.78	0.865	-0.52 (-6.64 to 5.60)

Table S4 reports post-exacerbation changes in health-related quality of life (HRQoL) among all patients with bronchiectasis exacerbations during follow-up. HRQoL was assessed using the Quality of Life Questionnaire-Bronchiectasis (QOL-B) v3.1, where higher QOL-B scores indicate better HRQoL. PRE and POST assessments correspond to the closest stable-state assessments before and after the first exacerbation. Mean ± SD interval from pre-exacerbation assessment to exacerbation was 49.18 ± 17.64 days and 52.91 ± 19.87 days from exacerbation to post-exacerbation assessment. Results are presented as mean (SD) for PRE and POST values, and as mean change (Δ = POST − PRE) with 95% confidence intervals (95%CIs). P-values were obtained using paired t-tests. *P <0.05.

Table S5. Post-exacerbation changes in QOL-B domains by colonisation status (n = 52).

QOL-B Domain	Colonisation status	PRE (mean ± SD)	POST (mean ± SD)	P-value	Delta (95% CI)
**Physical Functioning**	Intermittent	25.66 ± 22.14	35.26 ± 29.03	0.072	9.59 (-1.10 to 20.29)
	Chronic	48.83 ± 31.34	55.00 ± 29.72	0.154	6.16 (-2.42 to 14.75)
**Role Functioning**	Intermittent	40.03 ± 23.52	34.88 ± 35.16	0.633	-5.16 (-29.11 to 18.80)
	Chronic	64.15 ± 29.18	66.91 ± 27.77	0.383	2.77 (-3.58 to 9.12)
**Vitality**	Intermittent	40.28 ± 33.33	52.80 ± 17.25	0.352	12.52 (-16.71 to 41.75)‡
	Chronic	65.70 ± 27.03	61.54 ± 27.80	0.347	-4.16 (-12.99 to 4.67)
**Emotional Functioning**	Intermittent	65.75 ± 39.63	72.81 ± 25.22	0.549	7.06 (-18.93 to 33.04)‡
	Chronic	76.21 ± 23.98	76.26 ± 25.74	0.991	0.04 (-7.84 to 7.93)
**Social Functioning**	Intermittent	46.63 ± 31.90	45.04 ± 32.44	0.883	-1.58 (-25.66 to 22.49)
	Chronic	71.76 ± 32.05	62.89 ± 28.47	0.027*	-8.87 (-16.67 to -1.07)
**Treatment Burden**	Intermittent	76.31 ± 37.18	57.03 ± 26.82	0.277	-19.28 (-58.72 to 20.15)‡
	Chronic	78.65 ± 29.94	79.24 ± 24.33	0.909	0.60 (-9.91 to 11.10)
**Health Perceptions**	Intermittent	22.05 ± 21.96	20.06 ± 13.56	0.834	-1.99 (-23.14 to 19.15)
	Chronic	39.43 ± 21.35	38.64 ± 20.69	0.847	-0.79 (-9.03 to 7.45)
**Respiratory Symptoms**	Intermittent	77.29 ± 23.87	72.96 ± 24.33	0.670	-4.33 (-26.91 to 18.24)
	Chronic	73.12 ± 25.24	73.48 ± 21.50	0.906	0.36 (-5.82 to 6.54)

Table S5 shows post-exacerbation changes in health-related quality of life (HRQoL) according to the colonisation status (intermittent/chronic). HRQoL was assessed using the QOL-B v3.1, where higher scores indicate better HRQoL. PRE and POST assessments correspond to the closest stable-state assessments before and after the first exacerbation. The mean ± SD time from pre-exacerbation assessment to exacerbation was 49.18 ± 17.64 days and 52.91 ± 19.87 days from exacerbation to post-exacerbation assessment. Results are presented as mean ± SD for PRE and POST values and as mean change (Δ = POST − PRE) with 95%Cis, stratified by PA colonisation status (intermittent/chronic). P-values were obtained using paired t-tests. * P <0.05. ‡ differences exceeding the proposed minimum clinically important difference (MCID) threshold.

Table S6. Changes in QOL-B domains by *Pseudomonas aeruginosa* phenotype following the first exacerbation (n = 52).

QOL-B Domain	PA phenotype	PRE (mean ± SD)	POST (mean ± SD)	P-value	Delta (95% CI)
Physical Functioning	Non-mucoid	54.95 ± 30.15	50.85 ± 30.52	0.401	-4.10 (-14.01 to 5.80)
	Mucoid	47.32 ± 30.62	37.58 ± 30.68	0.078	-9.74 (-20.69 to 1.21)
Role Functioning	Non-mucoid	58.42 ± 32.23	58.94 ± 36.87	0.914	0.52 (-9.44 to 10.49)
	Mucoid	60.74 ± 27.48	62.71 ± 26.31	0.650	1.98 (-6.90 to 10.85)
Vitality	Non-mucoid	60.69 ± 30.07	60.66 ± 24.93	0.996	-0.03 (-14.46 to 14.39)
	Mucoid	61.16 ± 29.92	59.22 ± 27.85	0.720	-1.94 (-13.02 to 9.13)
Emotional Functioning	Non-mucoid	73.56 ± 24.34	67.90 ± 27.20	0.290	-5.65 (-16.44 to 5.13)
	Mucoid	83.98 ± 20.79	75.00 ± 30.88	0.091	-8.98 (-19.52 to 1.55)‡
Social Functioning	Non-mucoid	66.80 ± 34.33	59.70 ± 29.21	0.218	-7.10 (-18.70 to 4.51)
	Mucoid	67.27 ± 32.84	59.39 ± 30.80	0.121	-7.88 (-17.98 to 2.22)
Treatment Burden	Non-mucoid	75.04 ± 34.02	70.72 ± 26.91	0.639	-4.32 (-23.30 to 14.66)
	Mucoid	81.07 ± 28.06	79.89 ± 24.56	0.834	-1.18 (-12.69 to 10.33)
Health Perceptions	Non-mucoid	32.54 ± 21.01	32.00 ± 21.90	0.934	-0.54 (-13.88 to 12.80)
	Mucoid	39.51 ± 23.35	38.05 ± 19.66	0.721	-1.45 (-9.75 to 6.84)
Respiratory Symptoms	Non-mucoid	78.07 ± 25.94	77.12 ± 24.19	0.845	-0.96 (-10.96 to 9.05)
	Mucoid	70.06 ± 23.56	69.95 ± 19.17	0.976	-0.12 (-8.15 to 7.92)

Table S6 summarises post-exacerbation changes in health-related quality of life (HRQoL) according to *Pseudomonas aeruginosa* (PA) phenotype (non-mucoid/mucoid). HRQoL was assessed using the QOL-B v3.1, where higher scores indicate better HRQoL. PRE and POST assessments correspond to the closest stable-state assessments before and after the first exacerbation. The mean ± SD time from pre-exacerbation assessment to exacerbation was 49.18 ± 17.64 days and 52.91 ± 19.87 days from exacerbation to post-exacerbation assessment. Results are presented as mean (SD) for PRE and POST values and as mean change (Δ = POST − PRE) with 95%CIs, stratified by PA pgenotype (non-mucoid/mucoid). P-values were obtained using paired t-tests. ‡ differences exceeding the proposed minimum clinically important difference (MCID) threshold.

Table S7. Post-exacerbation changes in QOL-B domains by first exacerbation severity (n = 52).

QOL-B Domain	First exacerbation severity	PRE (mean ± SD)	POST (mean ± SD)	P-value	Delta (95% CI)
Physical Functioning	Ambulatory	30.32 ± 21.45	25.00 ± 20.72	0.425	-5.32 (-19.42 to 8.79)
	Hospitalised	58.29 ± 29.78	50.98 ± 31.33	0.095	-7.30 (-15.94 to 1.34)
Role Functioning	Ambulatory	66.07 ± 28.07	68.60 ± 30.25	0.508	2.53 (-5.13 to 10.19)
	Hospitalised	40.28 ± 26.07	37.82 ± 23.64	0.687	-2.45 (-15.51 to 10.60)
Vitality	Ambulatory	63.65 ± 30.53	60.58 ± 27.25	0.564	-3.07 (-13.78 to 7.63)
	Hospitalised	57.88 ± 23.85	52.78 ± 26.43	0.471	-5.10 (-20.14 to 9.93)
Emotional Functioning	Ambulatory	76.78 ± 25.42	76.14 ± 22.76	0.876	-0.64 (-8.96 to 7.68)
	Hospitalised	74.03 ± 33.29	66.67 ± 32.57	0.421	-7.36 (-26.76 to 12.04)
Social Functioning	Ambulatory	68.10 ± 33.83	61.15 ± 29.64	0.067	-6.94 (-14.40 to 0.51)
	Hospitalised	63.89 ± 32.44	54.70 ± 30.79	0.378	-9.19 (-31.20 to 12.81)‡
Treatment Burden	Ambulatory	77.05 ± 29.83	77.42 ± 23.32	0.951	0.37 (-11.89 to 12.63)
	Hospitalised	81.82 ± 34.52	70.42 ± 32.66	0.259	-11.40 (-32.61 to 9.82)‡
Health Perceptions	Ambulatory	39.89 ± 22.18	36.83 ± 21.22	0.482	-3.06 (-11.80 to 5.68)
	Hospitalised	30.11 ± 19.30	25.00 ± 19.46	0.487	-5.11 (-20.77 to 10.54)
Respiratory Symptoms	Ambulatory	75.39 ± 24.43	74.15 ± 20.34	0.690	-1.23 (-7.45 to 4.99)
	Hospitalised	71.06 ± 26.53	69.44 ± 26.43	0.848	-1.62 (-19.78 to 16.54)

Table S7 summarises post-exacerbation changes in health-related quality of life (HRQoL) according to the severity of the first bronchiectasis exacerbation (ambulatory/hospitalised). HRQoL was assessed using the QOL-B v3.1, where higher scores indicate better HRQoL. PRE and POST assessments correspond to the closest stable-state assessments before and after the first exacerbation. The mean ± SD time from pre-exacerbation assessment to exacerbation was 49.18 ± 17.64 days and 52.91 ± 19.87 days from exacerbation to post-exacerbation assessment. Results are presented as mean (SD) for PRE and POST values and as mean change (Δ = POST − PRE) with 95%Cis, stratified by severity of the first exacerbation (ambulatory/hospitalised). P-values were obtained using paired t-tests. ‡ differences exceeding the proposed minimum clinically important difference (MCID) threshold.
